# Tuberculosis: Clinical Laboratory Diagnostic Techniques and Future Perspectives

**DOI:** 10.3390/vaccines14010038

**Published:** 2025-12-29

**Authors:** Qiuyue Song, Junlin Liu, Chunhua Wang

**Affiliations:** 1 Department of Clinical Laboratory, Xiangyang No. 1 People’s Hospital, Hubei University of Medicine, Xiangyang 441000, China; songqiuyue2025@163.com; 2 Department of Clinical Laboratory, Xiangyang Central Hospital, Affiliated Hospital of Hubei University of Arts and Science, Xiangyang 441021, China; liujl20240101@163.com; 3 Hubei Shizhen Laboratory, School of Laboratory Medicine, Hubei University of Chinese Medicine, Wuhan 430065, China

**Keywords:** tuberculosis, *Mycobacterium tuberculosis*, laboratory diagnostic techniques

## Abstract

Tuberculosis is a severe infectious disease caused by *Mycobacterium tuberculosis* (MTB) infection and poses a serious public health challenge globally. The prevalence of multidrug-resistant MTB in countries with a high burden of tuberculosis has further increased the challenges of tuberculosis prevention and control. The rapid and accurate diagnosis of MTB and multidrug-resistant MTB serves as the prerequisite and key to controlling tuberculosis transmission and prevalence. However, the insufficient laboratory diagnosis capacity of tuberculosis seriously constrains the detection of tuberculosis cases, leading to delayed treatment and interpersonal transmission. Although multiple laboratory diagnostic techniques for tuberculosis have emerged, their diagnostic efficacy varies significantly. This review conducts a detailed analysis of the principles, characteristics, and clinical applications of various laboratory diagnostic techniques across three major categories: bacteriological morphology, molecular biology, and immunology. It elucidates the advantages and disadvantages of each technique and explores future development directions for tuberculosis laboratory diagnostics, aiming to provide valuable methodological references for the clinical diagnosis and treatment of tuberculosis.

## 1. Background

Tuberculosis is a chronic infectious disease caused by *Mycobacterium tuberculosis* (MTB) infection, predominantly found in Southeast Asia, Africa, and the Western Pacific regions. It ranks as the second most fatal disease caused by a single infectious agent (second only to COVID-19), posing a serious threat to human life and global public health security [1]. According to the World Health Organization (WHO) estimates, approximately 10.8 million people worldwide contracted tuberculosis in 2023 [2]. Early and precise diagnosis of tuberculosis, along with timely and effective treatment, is crucial for reducing tuberculosis morbidity and mortality.

Insufficient capacity for tuberculosis laboratory diagnosis represents a major obstacle to achieving the 2035 “End Tuberculosis” goal, seriously hampering the timely detection of tuberculosis patients and leading to missed diagnosis, treatment delays, and interpersonal transmission of tuberculosis. The development of rapid, precise, and cost-effective laboratory diagnostic techniques for tuberculosis is essential for controlling the epidemic. Traditional etiological diagnostic techniques for tuberculosis primarily include sputum smear microscopy, sputum smear fluorescence staining techniques, and MTB culture. Although these methods have played a significant role in tuberculosis diagnosis, they exhibit limitations in sensitivity and specificity, require prolonged turnaround time, and present deficiencies in diagnosing latent tuberculosis, tuberculosis complicated with other diseases, and drug-resistant tuberculosis [3,4]. Furthermore, these traditional diagnostic techniques have constrained early identification of tuberculosis patients and rapid initiation of treatment decisions, thereby further exacerbating interpersonal transmission and mortality rates associated with tuberculosis [5]. With the development of modern diagnostic techniques, various molecular biology and immunology diagnostic techniques have been progressively applied in the field of tuberculosis diagnosis. These primarily include PCR detection technology, isothermal amplification technology, gene chip technology, enzyme-linked immunospot assay (ELISPOT), demonstrating significant potential in enhancing diagnostic sensitivity, shortening turnaround time, and enabling early identification of drug-resistant MTB [6,7,8,9]. Additionally, the new generation of molecular diagnostic technologies based on CRISPR/Cas systems and high-throughput sequencing are emerging in the tuberculosis diagnosis field, characterized by high sensitivity, specificity, portability, and rapid response capabilities [10].

The diverse diagnostic techniques for tuberculosis exhibit varying diagnostic efficacy, posing significant challenges to the clinical diagnosis and treatment of tuberculosis. This study analyzed the bacteriological morphology, molecular biology, and immunological diagnostic techniques for tuberculosis in terms of technical principles, characteristics, and clinical applications in detail. Meanwhile, it discussed the advantages and disadvantages of various techniques and the future development directions for tuberculosis laboratory diagnostic techniques. This review aims to provide valuable references for clinical decision-making regarding tuberculosis diagnostic technologies (Table 1).

## 2. Bacterial Morphological Diagnostic Techniques for Tuberculosis

MTB, the causative agent of tuberculosis, is a slender and slightly curved bacillus transmitted via the respiratory tract. The morphological identification of MTB is a commonly used method for tuberculosis diagnosis, including microscopic observation and bacteriological culture morphological diagnostic techniques. These techniques, with their long history and extensive application, play significant roles in the clinical diagnosis of tuberculosis.

### 2.1. Sputum Smear Microscopy Examination Technique

**Figure 1:**
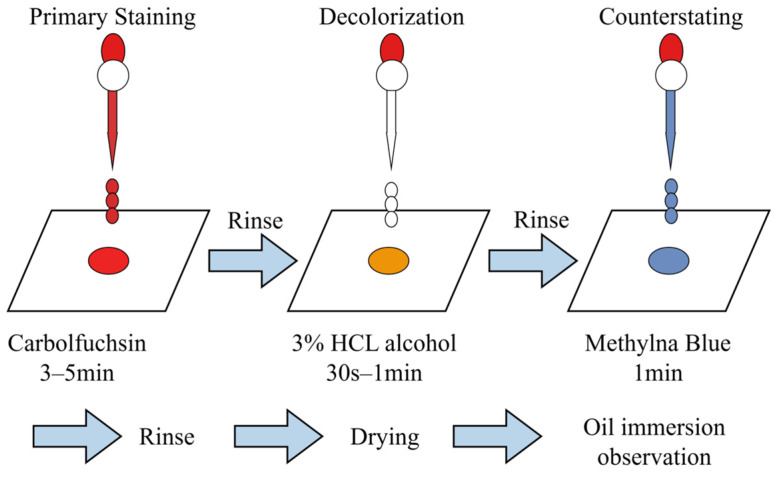
Schematic diagram of the Z-N acid-fast staining.

Sputum smear microscopy examination is the most widely used traditional technique in the clinical laboratory diagnosis for tuberculosis, primarily including the Ziehl-Neelsen (Z-N) acid-fast staining and fluorescent staining (such as Auramine O staining) methods. MTB is not decolorized by acid–alcohol after staining (Figure 1), exhibiting acid-fast properties, which may be related to its cell wall being rich in mycolic acids. Following sputum smear acid-fast staining, bacteria exhibiting acid-fast characteristics (red-stained bacilli) can be observed under an ordinary optical microscope using an oil immersion lens (100×), which can provide a preliminary identification of MTB. The acid-fast staining method for sputum smears is characterized by low cost, simple operation, and rapid results. It is suitable for preliminary screening diagnosis of pulmonary tuberculosis patients, particularly in resource-limited settings, where this technique serves as one of the primary methods for clinical laboratory diagnosis of tuberculosis. However, the sensitivity of acid-fast staining is relatively low, generally around 50–60% [11], particularly in sputum samples with low bacterial loads which are more likely to cause missed diagnosis. After acid-fast bacilli are stained with fluorescent dye (Auramine O), golden-yellow fluorescent bacilli can be observed under fluorescence microscopy (such as the LED fluorescence microscope recommended by WHO), allowing for preliminary identification of MTB. This method enables observation under low-power magnification, facilitating rapid detection of acid-fast bacilli and reducing sample testing turnaround time. Furthermore, the use of fluorescent dyes has significantly improved diagnostic sensitivity, reaching 70–80% [11,12]. The sputum smear fluorescent staining method has gradually been adopted in the clinical laboratory diagnosis of tuberculosis, but the cost is substantially higher than that of acid-fast staining.

It is noteworthy that since nontuberculous mycobacteria (NTM) are also acid-fast [13], neither sputum smear acid-fast staining nor sputum smear fluorescent staining can differentiate between MTB and NTM. Moreover, the sputum smear staining cannot detect bacterial drug-resistance and requires combined usage with bacterial culture or molecular diagnostic techniques to improve diagnostic accuracy. In recent years, sputum smear staining-microscopy detection technology has been continuously refined. For example, pretreating samples with 1% bleach before staining can significantly increase the positive rate of clinical diagnosis for MTB [14]. Furthermore, fluorescence microscopy technology utilizing specific DNA aptamers to label MTB has enhanced detection sensitivity and specificity [15]. The integration of automated microscopy systems with artificial intelligence technology has also enhanced the efficiency and accuracy of sputum smear staining-microscopy examination techniques [16,17]. In summary, sputum smear staining-microscopy examination techniques, valued for their simplicity and rapidity, remain a critical method for tuberculosis diagnosis in clinical laboratories. Widely applicable in both clinical and laboratory settings, they play an irreplaceable role particularly in resource-limited areas [11,12].

### 2.2. Bacterial Culture Techniques

The MTB culture technique serves as the gold standard for tuberculosis diagnosis in clinical laboratories and primarily includes solid and liquid culture methods. MTB typically requires 6–8 weeks to develop characteristic opaque, buff-colored, rough, dry, raised cauliflower-like colonies on Lowenstein–Jensen (L-J) solid culture medium, exhibiting good specificity. Additionally, comparative culture using selective media containing p-nitrobenzoic acid (PNB) and thiophene-2-carboxylic acid hydrazide (TCH) allows accurate differentiation between MTB and NTM through the observation of distinct colonial growth characteristics, ensuring diagnostic accuracy [11]. Although the solid culture environment is stable and facilitates the morphological observation of colonies, this technique is seriously limited in application for clinical diagnosis of tuberculosis. The most apparent drawback of solid culture technology is that MTB grows relatively slowly on solid culture medium with a prolonged cultivation period, which leads to delays in diagnosis and subsequent treatment of tuberculosis patients [18]. Additionally, the solid culture process is more susceptible to contamination, compromising the accuracy of cultivation results [19].

In contrast, the liquid culture technique often demonstrates an absolute advantage in rapid cultivation. Liquid culture enables us to detect the growth of MTB several days to several weeks earlier than solid culture, such as the BACTEC MGIT 960, which requires only 10–20 days to detect MTB [18,20]. The BACTEC MGIT 960, a fully automated mycobacterial culture monitoring system, is a rapid culture system based on liquid culture technology, integrating rapid cultivation, detection, and drug susceptibility testing of mycobacteria. By continuously monitoring changes in fluorescence intensity within the culture tube to reflect the amount of oxygen consumed in the liquid culture medium for determining the mycobacterial growth. This cultivation system automatically detects fluorescent signals within the culture tube every 60 min, features an automatic alert function, significantly improves detection efficiency, and demonstrates superior sensitivity compared with solid culture techniques, markedly enhancing the positive detection rate of MTB [11,21,22]. The BACTEC MGIT 960 system achieved a sensitivity of 96% for MTB complex detection, typically around 90%, whereas solid culture methods generally yield sensitivities ranging between 75% and 80% [23,24,25]. However, liquid culture exhibits slightly lower specificity than solid culture. Furthermore, the equipment, maintenance, and reagent costs associated with liquid culture systems are substantial, leading to difficult implementation in resource-limited, high-burden tuberculosis areas [26,27]. Liquid culture technique still faces an inevitable contamination issue, though it shows improvement compared to solid culture techniques [28].

In recent years, researchers have attempted to overcome technical barriers in existing culture methods by developing novel biphasic media for mycobacteria. By integrating the advantages of solid and liquid culture technologies, these innovations maintain the high specificity of solid cultures while enhancing the sensitivity and incubation speed of liquid cultures [29]. Previous studies have demonstrated that the biphasic culture exhibits a higher recovery rate than the L-J culture medium (*p* < 0.001), with performance similar to that of MGIT 960 in sputum smear-positive specimens. The median time to positive for biphasic culture was 20 days, representing a 10-day reduction compared to Roche solid culture [29]. The biphasic culture offers advantages over traditional solid culture methods, including higher positive detection rates and shortened turnaround time, demonstrating promising potential for clinical applications. In brief, the MTB culture remains the gold standard for tuberculosis diagnosis with high specificity and accuracy, while the long culture period, stringent culture medium requirements, and contamination risks limit its utility in clinical rapid diagnosis and treatment monitoring of tuberculosis.

## 3. Molecular Biology Diagnosis Techniques for Tuberculosis

Tuberculosis molecular biology diagnostic techniques are a category of methods that utilize MTB DNA as a template and perform target detection based on the PCR principle. These primarily include nested PCR technology, isothermal amplification technology, and gene chip technology. These techniques exhibit high sensitivity and specificity, rapidly obtain the detected results, and simultaneously identify drug-resistant genes, demonstrating significant application value in the clinical diagnosis and treatment of tuberculosis.

### 3.1. Nested PCR Technique

**Figure 2:**
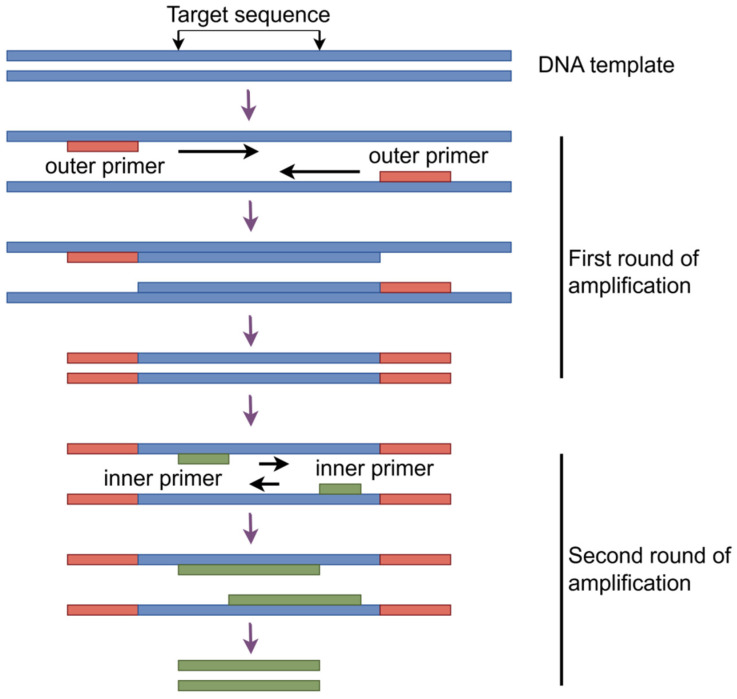
Schematic diagram of nested PCR amplification.

Nested PCR is a modified gene amplification technique, which enhances the accuracy of detection results by performing two rounds of amplification on the target gene using two pairs of specific primers (Figure 2). For tuberculosis diagnosis, nested PCR has been widely applied in the detection of MTB in clinical samples. Compared to conventional PCR techniques, the detection limit of nested PCR is as low as 1 pg/μL [30,31], significantly improving the detection sensitivity. Simultaneously, the nested primer design effectively reduces non-specific amplification and false positive results, enhancing the specificity of detection outcomes, particularly excelling in clinical samples with complex backgrounds [32]. These characteristics give it significant potential for application in the diagnosis of tuberculosis patients with clinical low bacterial loads infections. Nested PCR primers constructed with the hsp65 gene of the MTB standard strain H37Rv have a detection limit as low as 0.3 pg and exhibit high specificity, exhibiting no cross-reactivity with nontuberculous mycobacteria, other bacteria, and fungi [33]. Furthermore, nested PCR has demonstrated strong adaptability to various sample types, covering sputum, blood, brain tissue, urine, intraocular fluid, and ascites. This versatility gives it broad application value in pulmonary tuberculosis and various forms of tuberculosis (such as genitourinary tuberculosis, brain tuberculosis, and abdominal tuberculosis) [31,34,35]. However, nested PCR also has certain limitations. The operational procedure is relatively cumbersome, requiring two rounds of amplification. This extends the detection turnaround time and increases contamination risks. Particularly, cross-contamination may cause false positives, necessitating strict laboratory operating protocols and separate work area management [32]. Most importantly, nested PCR cannot provide information about the viability status of strains or replace culture-based drug susceptibility testing, and it also has limited capability for detecting genetic mutations. Therefore, it must be combined with other molecular methods for comprehensive drug resistance analysis [36,37].

Xpert MTB/RIF is a molecular diagnostic system developed based on modified nested PCR technology, enabling simultaneous detection of MTB and rifampicin resistance genes. The system employs a fully automated cartridge-based kit throughout the process, which minimizes sample contamination risk and operational complexity, making it suitable for rapid diagnosis in resource-limited settings. Compared with traditional microscopy and culture methods, Xpert MTB/RIF exhibits significant advantages, such as rapid detection (approximately 2 h), high sensitivity and specificity, user-friendly operation, and no requirement for high-level laboratory facilities [38,39]. Notably, this system exhibits different detection sensitivity across different types of clinical specimens. Higher detection sensitivity has been observed in respiratory tract samples (such as sputum and bronchoalveolar lavage fluid), while reduced sensitivity occurred in peripheral body fluids or paucibacillary samples (such as urine, cerebrospinal fluid, lymph node aspirates) [40]. The latest upgraded Xpert MTB/RIF Ultra system signifi1cantly enhances detection capability for paucibacillary tuberculosis infections by combining nested PCR with molecular beacon technology and incorporating IS6110 and IS1081 detection targets. This advancement substantially reduced the detection limit and improved positive diagnosis rates, particularly demonstrating superior diagnostic performance in smear-negative pulmonary tuberculosis, pediatric tuberculosis, and extrapulmonary tuberculosis (e.g., tuberculous meningitis) [38,39,41]. Previous studies have demonstrated that Xpert MTB/RIF Ultra exhibits higher sensitivity than Xpert MTB/RIF, as Xpert MTB/RIF Ultra achieved a sensitivity of 90.9% (86.2–94.7%), whereas Xpert MTB/RIF showed 84.7% (78.6–89.9%) sensitivity for pulmonary tuberculosis diagnosis [42]. Moreover, the sensitivity of Xpert MTB/RIF Ultra reached 90.91%, which was significantly higher than that of Xpert MTB/RIF (78.79%) for bone and joint tuberculosis diagnosis [43]. The Xpert MTB/RIF system, characterized by its rapidity, automation, and high accuracy, has been recommended by the WHO as the preferred molecular diagnostic method for rapid diagnosis of tuberculosis and rifampicin resistance, providing robust technical support for precision diagnosis and public health control of tuberculosis. However, it still has limitations in distinguishing between live and dead bacteria, potentially leading to false positives due to residual DNA, which may affect the diagnosis of patients undergoing retreatment.

### 3.2. Isothermal Amplification Technique

**Figure 3:**
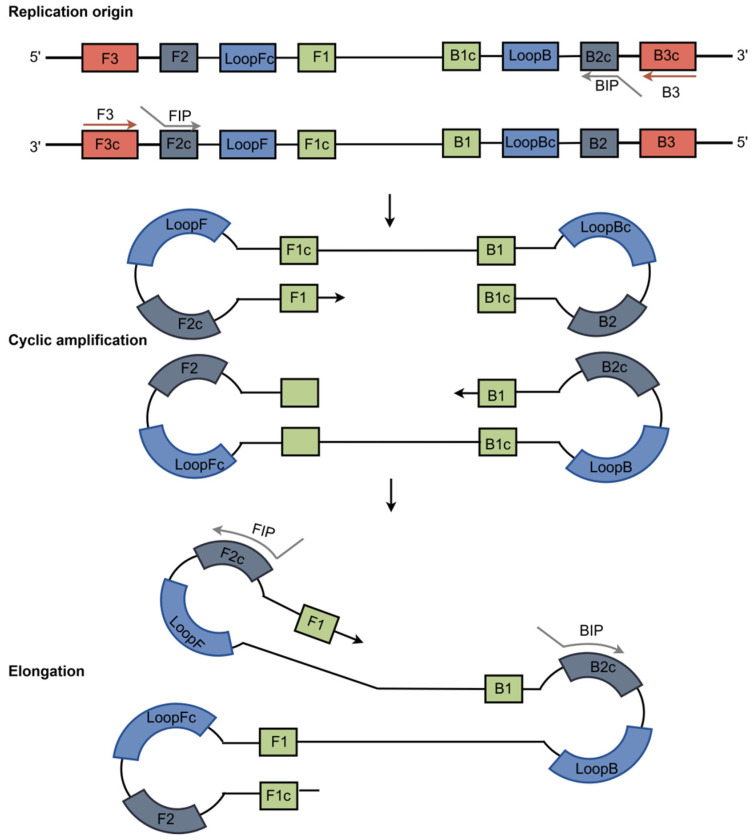
Schematic diagram of LAMP (FIP and BIP are inner primers, B3 and F3 are outer primers).

Isothermal amplification technique is a molecular diagnostic technique that amplifies genes in vitro under constant temperature conditions. It demonstrates great potential in tuberculosis diagnosis due to its simple operation, elimination of expensive thermal cycling instruments, rapid reaction time, and high sensitivity. Loop-mediated isothermal amplification (LAMP) technology currently is one of the most extensively researched and applied isothermal amplification techniques. Typically conducted at constant temperatures of 60–65 °C, it utilizes 4 or 6 specific primers and Bst DNA polymerase to rapidly amplify target sequences in large quantities through strand displacement reactions (Figure 3), completing the amplification process within 30–60 min [44,45]. LAMP technology not only exhibits high specificity and sensitivity but also enables visual interpretation of results through color changes or fluorescence signal intensity of amplified products, greatly facilitating its application in primary care and remote areas [46,47].

Multiplex LAMP technology further enhances the accuracy of tuberculosis laboratory diagnosis. In particular, multiplex LAMP technology has demonstrated high sensitivity and specificity for extrapulmonary tuberculosis diagnosis based on targeting multiple genes (*mpt64*, *IS6110*, *pstS1*), such as abdominal tuberculosis (sensitivity: 84.6%, specificity: 97.3%), bone and joint tuberculosis (sensitivity: 82.4%, specificity: 96.9%), and genitourinary tuberculosis (sensitivity: 85.5%, specificity: 94.4%) [48,49,50]. This performance was superior to single-target LAMP and significantly better than conventional PCR and bacterial culture methods. Additionally, reagents for LAMP detection are prepared through lyophilization or air-drying processes, enabling stable storage at room temperature for several months, significantly enhancing the practicality of field testing [51]. Furthermore, LAMP detection can rapidly identify drug resistance gene mutations in MTB, enabling early diagnosis of multidrug-resistant (MDR) and extensively drug-resistant (XDR) tuberculosis, providing reliable evidence for targeted therapy [52,53]. Although LAMP technology demonstrates multiple advantages in tuberculosis diagnosis, its false-positive issues in clinical applications cannot be overlooked. Several studies have indicated that LAMP testing may also yield false-negative results, necessitating comprehensive evaluation in conjunction with clinical manifestations or supplementary diagnostic techniques [54]. To date, isothermal amplification technology, particularly LAMP, has become an essential tool for molecular diagnosis of tuberculosis due to its rapidity, simplicity, high sensitivity, and low equipment requirements. Its widespread application in resource-limited regions contributes to improving early diagnosis rates for tuberculosis, facilitating timely treatment, and containing the spread of tuberculosis [44,55].

### 3.3. Gene Chip Technique

The gene chip technique relies on specific oligonucleotide probes fixed on chip carriers to capture target sequences, enabling rapid identification of key gene mutant sites in MTB and efficient screening for strain identification and drug resistance genes. This technology has gained widespread attention in the laboratory diagnosis of multidrug-resistant MTB. Compared with conventional bacterial culture and drug susceptibility testing, gene chip technology significantly reduces detection time while enhancing sensitivity and specificity, making it particularly suitable for early diagnosis of drug-resistant tuberculosis and epidemiological surveillance. Traditional drug susceptibility testing typically requires several weeks, whereas gene chip detection delivers results within hours to one day, substantially improving the efficiency of clinical diagnosis and therapeutic decision-making [56]. Gene chip technology also demonstrates significant value in identifying atypical tuberculosis and NTM infections. A study on rapid NTM detection in bronchoalveolar lavage fluid revealed that gene chip technology achieves comparable positive rates to traditional liquid culture methods, with species identification accuracy reaching to 96.79%, providing a rapid approach for early diagnosis and treatment of NTM pulmonary disease [57]. Additionally, this technique can also achieve rapid identification of MTB in peripheral tissue biopsy specimens, promoting early diagnosis and treatment of tuberculosis in sites such as the larynx [58]. With advances in chip manufacturing technology and molecular biology methods, microfluidic chip systems integrating PCR amplification and chip hybridization have achieved high-throughput and high-sensitivity nucleic acid detection, providing new development directions for tuberculosis diagnostic techniques [59]. However, the diagnostic performance of gene chip technology is significantly affected by sample type and bacterial loads, particularly presenting a risk of missed detection in samples with low bacterial loads [60].

### 3.4. Emerging Molecular Diagnostic Techniques

There are some other emerging molecular detection methods applied in tuberculosis laboratory diagnosis recently, such as the line probe assay (LPA), CRISPR/Cas-based detection technology, and whole genome sequencing (WGS). These methods exhibit unique advantages in enhancing diagnostic speed, accuracy, and drug resistance detection, providing crucial support for precise tuberculosis diagnosis and treatment.

LPA is a molecular detection technique based on specific oligonucleotide probe hybridization, enabling rapid detection of MTB and drug resistance gene mutations against anti-tuberculosis drugs (particularly rifampicin and isoniazid). LPA achieves rapid screening of drug resistance mutant sites by amplifying target gene fragments, followed by hybridization with specific probes on the membrane. Compared to traditional culture, this technique offers a shorter detection cycle and higher sensitivity, making it particularly suitable for diagnosing MDR and XDR tuberculosis. Comparative studies on the detection performance of LPA, WGS, and conventional drug susceptibility testing (DST) revealed that LPA demonstrated high specificity (approaching 100%) in detecting rifampicin and isoniazid resistance, while its sensitivity was slightly lower than that of WGS. Particularly in isoniazid resistance detection, LPA exhibited insufficient sensitivity, indicating that, while it enables rapid screening of common mutations, its capacity to detect certain rare or novel mutations remains limited [61]. Although LPAs are relatively simple to operate, suitable for resource-limited settings, and recommended by the WHO for initial screening of drug-resistant tuberculosis, they have certain limitations. For instance, probes are designed only for known hotspot drug resistance gene mutations, which cannot cover all drug resistance mechanisms. In addition, they require a sufficient bacillary load in samples, resulting in lower detection sensitivity for sputum smear-negative samples.

The CRISPR/Cas system is an emerging technology integrating gene editing and molecular detection, which has recently been introduced into the field of molecular diagnosis for tuberculosis. This technology utilizes the highly specific recognition and cleavage functions of CRISPR-associated proteins on target DNA sequences to achieve rapid detection of MTB-specific genes. The CRISPR/Cas system can accurately identify and amplify targets, triggering the trans-cleavage ability of effector proteins Cas12a or Cas13a to cut reporter single-stranded DNA (ssDNA) molecules after recognizing the target. This technology combines the dual advantages of target amplification and signal amplification, enhancing the sensitivity and specificity of detection techniques. Studies have shown that the RAA-CRISPR-Cas12a technology targets the detection of the *MTB IS6110* gene, with a sensitivity of 3.13 CFU/mL. Clinical sample testing validated its clinical detection sensitivity and specificity at 88.3% and 94.0%, respectively, with an area under the ROC curve of 0.944, an overall detection time of less than 1.5 h, ease of operation and no requirement for complicated equipment, making it suitable for rapid clinical screening and applications in resource-limited environments [62]. Clinical sample verification has shown that the ERA-CRISPR/Cas12a detection technology has a 100% consistency with commercial qPCR, and its detection limit is as low as nine copies/μL. The detection time is approximately 50 min, and it shows no cross-reaction with non-tuberculous mycobacteria, demonstrating excellent sensitivity and specificity [63]. The CRISPR/Cas13a system achieves highly sensitive detection by targeting the specific RNA sequences of MTB, with a detection limit as low as one copy/μL. It shows superior diagnostic performance compared to traditional acid-fast staining and bacterial culture in clinical samples such as sputum, bronchoalveolar lavage fluid, and pus, comparable to GeneXpert MTB/RIF detection results, making it especially useful for quick diagnoses in places with limited resources [64]. Additionally, electrochemical biosensors based on CRISPR/Cas12a have been developed for the detection of MTB-specific DNA, demonstrating rapid detection (in about 60 min) without the need for pre-amplification and with a sensitivity of 14.5 nM, expanding the application scenarios of the CRISPR/Cas system in tuberculosis diagnosis [65]. To further enhance the convenience and accuracy of detection, chain hybridization-based CRISPR-lateral flow assay (CRISPR-CHLFA) technology has been developed. This method replaces traditional immunoassays with nucleic acid hybridization, significantly reducing the false-positive rate and enabling highly accurate visual detection of MTB target genes, with clinical sample analysis achieving 100% accuracy, providing a new approach for point-of-care testing [66]. Meanwhile, the creation of multiplex CRISPR/Cas platforms, such as a dual-channel detection system combining Cas12a and Cas13, has enabled simultaneous detection of MTB and respiratory syncytial virus, showing greater potential for using CRISPR technology in multiplex detection [67].

WGS is a high-resolution molecular detection method that can comprehensively analyze all sequence information in the MTB genome, particularly demonstrating unique advantages in detecting mutations related to drug resistance. The WGS technology sequences the entire genome, identifying both known and unknown mutations associated with drug resistance, providing precise resistance profiles that support personalized treatment plans. Previous studies have analyzed drug-resistant MTB strains using WGS, discovering various combinations of resistance-related mutations that can accurately predict the phenotypes of multidrug-resistant (MDR) and extensively drug-resistant (XDR) tuberculosis [68]. Additionally, WGS can reveal differences in the number and distribution of resistance mutations, which helps in understanding the complexity of resistance mechanisms and their effects on drug sensitivity [69]. WGS is not limited to resistance detection; it can also be used to track the dynamics of tuberculosis transmission. By comparing the genomic sequences of different clinical isolates, WGS can identify the genetic distances between strains. It can also infer transmission chains and outbreak events, and it can reveal the populations of drug-resistant tuberculosis and their epidemiological characteristics, indicating that local drug-resistant strains often arise from recent transmissions, emphasizing the importance of interrupting transmission chains [70,71]. Furthermore, WGS can assist public health decision-making; for instance, in a study in Oman, WGS revealed the transmission pathways of tuberculosis between foreign workers and local residents, providing scientific evidence for the development of targeted prevention and control policies [72]. However, there are still many challenges to the widespread use of WGS in clinical settings. Firstly, sequencing costs are high, especially in low- and middle-income countries with limited resources, which restricts the dissemination of this technology [73]. Secondly, the data analysis process is complex, requiring specialized bioinformatics support and standardized analytical processes; currently, there is no globally unified standard, making the interpretation and comparison of results challenging [74,75]. Additionally, conducting WGS directly from clinical samples is still limited by the low content of pathogen DNA in the samples and interference from host and other microbial DNA. While there have been attempts using enrichment technologies such as microfluidics, more optimization is still needed [76,77]. Although WGS has superior detection capabilities for mixed infections compared to traditional methods, its sensitivity is still affected by factors such as sequencing depth [78].

## 4. Immunological Diagnostic Techniques for Tuberculosis

Immunological diagnostic techniques for tuberculosis are a series of technologies developed based on humoral and cellular immune responses, including tuberculosis antibody detection, tuberculin skin test (TST), enzyme-linked immunospot assay (ELISPOT), and interferon-gamma release assays (IGRAs). These techniques demonstrate distinct advantages in the diagnosis of latent tuberculosis infection.

### 4.1. Traditional Immunological Diagnostic Techniques

Tuberculosis antibody detection and the TST represent two classical immunological diagnostic techniques for tuberculosis. Antibody detection primarily targets MTB-specific antigens, assisting in determining infection status or disease progression by measuring antibody levels in serum or other biological samples of tuberculosis patients. However, current antibody detection methods still exhibit limitations in sensitivity and specificity, requiring further optimization and validation. Currently available commercial rapid antibody tests, such as the NOVA rapid total antibody test for tuberculosis, demonstrate suboptimal performance, with a sensitivity of only about 40% for diagnosing active pulmonary tuberculosis and even lower detection sensitivity for latent infection, limiting their clinical application value [79]. Nevertheless, studies on antibody reactivity to different protein antigens of MTB have revealed that certain antigens possess favorable antibody recognition capabilities. It was found that the IgG levels against major MTB-secreted proteins Rv1860 and Ag85B were significantly elevated in patients. Furthermore, the area under the ROC curve (AUC) for detection using native proteins exceeded 0.81, demonstrating promising diagnostic potential [80]. The optimization of antigen selection and innovations in detection platforms are expected to enhance the early diagnosis rates of tuberculosis and the ability to assist in disease assessment.

TST, as a vital tool for diagnosing MTB infection, has a long history of use and an extensive foundation in clinical practice. TST involves intradermal injection of tuberculin to detect the delayed-type hypersensitivity reaction to MTB-related antigens, thereby indicating whether the host is infected with MTB. The application and interpretation of TST results are influenced by multiple factors, including the history of Bacillus Calmette–Guerin (BCG) vaccination (false positive response from TST for patients previously vaccinated against MTB), NTM infections, immune status, and age [81], which imposes certain limitations on its specificity and sensitivity. However, this technique features low cost and simple operation, remaining the primary screening method for tuberculosis in many regions worldwide.

### 4.2. ELISPOT

**Figure 4:**
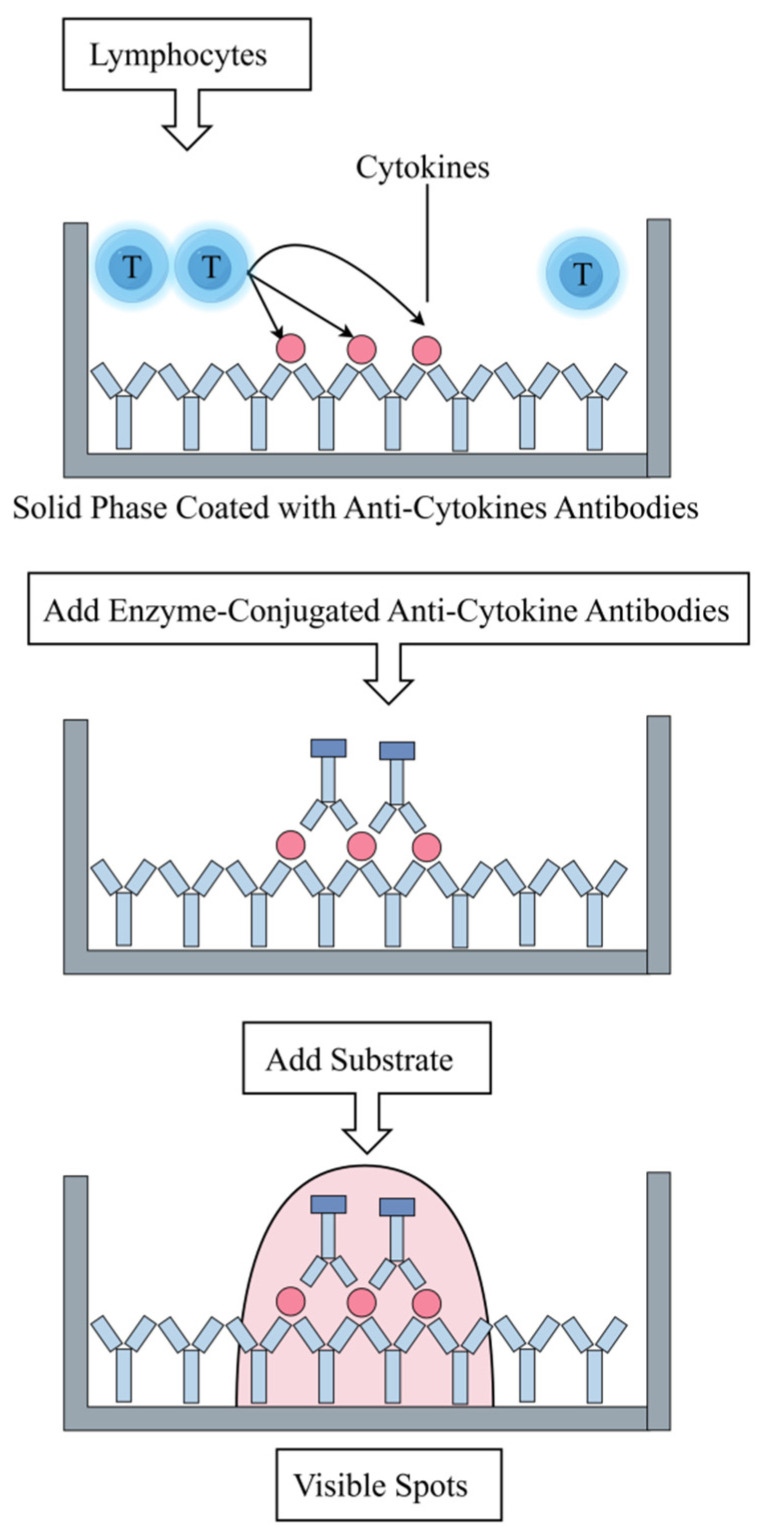
Schematic diagram of the principle of ELISPOT detection.

ELISPOT is a high-sensitivity immunological detection technique capable of detecting the immune activity of sensitized cells at the single-cell level, particularly the production of cytokines. ELISPOT is primarily used to detect T-cell responses to MTB-specific antigens by measuring secreted interferon-γ (IFN-γ), indirectly reflecting the quantity of MTB-sensitized T lymphocytes in the body (Figure 4). This technique not only aids in distinguishing between active and latent infections but also plays a crucial role in epidemiological investigations and contact tracing [82].

In clinical applications, the diagnostic value of ELISPOT for tuberculosis has been widely demonstrated. A rapid diagnostic study on pulmonary tuberculosis recurrence found that MTB-specific ELISPOT detection using bronchoalveolar lavage fluid demonstrated significantly higher sensitivity than Xpert MTB/RIF molecular testing, particularly exhibiting superior diagnostic accuracy in patients with recurrent paucibacillary pulmonary tuberculosis [83]. Since BCG lacks the ESAT-6 and CFP-10 encoding gene sequences, ELISPOT detection quantifies sensitized T lymphocytes stimulated by MTB-specific antigens (ESAT-6, CFP-10, and their fusion proteins), enabling differentiation between BCG-vaccinated individuals and tuberculosis-infected patients [84]. ELISPOT technology demonstrates unique advantages in the stratified diagnosis of tuberculosis patient populations. In pediatric tuberculosis diagnosis, T-SPOT.TB (based on ELISPOT technology) shows high sensitivity and specificity, facilitating early diagnosis [85]. Among immunocompromised populations such as diabetic patients, the IFN-γ response detected by ELISPOT in bronchoalveolar lavage fluid remains unaffected by this condition, exhibiting more stable immune response detection capability compared to peripheral blood ELISPOT [86]. Notably, ELISPOT is not limited to tuberculosis diagnosis but is also widely applied in tuberculosis vaccine development and immune mechanism research [87,88]. However, ELISPOT testing also has certain limitations, such as requiring a high-standard operational environment and needing to consider factors such as antigen selection, cell viability, and sample collection timing. In the future, novel ELISPOT platforms with simplified procedures and reduced detection time, along with technologies such as FluoroSpot, which combine multiple cytokine detection, are expected to further enhance the diagnostic efficiency and accuracy for tuberculosis [89,90].

ELISPOT, as a sensitive and specific immunological detection technique, plays a crucial role in laboratory diagnosis of tuberculosis. By detecting IFN-γ secretion from MTB-specific T cells, ELISPOT not only facilitates early diagnosis of active and latent infections but also aids in differentiating recurrent cases, guiding clinical treatment and vaccine development. It constitutes an essential component of tuberculosis diagnostic techniques [82,83,91].

### 4.3. IGRA

IGRA is an immunological detection technique that indirectly reflects MTB infection by measuring levels of IFN-γ released from T cells after stimulation with MTB-specific antigens. IGRA plays a crucial role in the diagnosis of latent tuberculosis infection (LTBI) and active tuberculosis and is increasingly being utilized in screening management within TB-endemic regions and high-risk populations. It demonstrates superior capability over TST in predicting progression from LTBI to active tuberculosis [92]. IGRA is not only employed for LTBI screening, but it can also serve as an auxiliary diagnostic tool for active tuberculosis. Patients with active tuberculosis exhibit significantly higher levels of IFN-γ secretion compared to individuals with latent infection [93,94]. Multiple studies have confirmed that IGRA offers distinct advantages in populations with a history of BCG vaccination, immunocompromised individuals, and other high-risk groups by reducing false-positive rates and optimizing resource allocation [92,95]. IGRA demonstrates excellent specificity and sensitivity, particularly excelling in detecting latent tuberculosis infection and predicting its progression to active disease. However, its high cost limits its widespread clinical application.

## 5. Imaging and Computer-Aided Diagnosis (CAD)

### 5.1. Traditional Imaging Diagnosis Methods

Chest X-rays and CT scans are important imaging tools for the diagnosis of tuberculosis, helping assess lung lesions. Chest X-rays quickly display typical tuberculosis lesions, helping make initial assessments; CT scans offer more detailed information about lesions, especially helpful for early detection and telling it apart from other diseases. However, traditional imaging can be subjective and has its limitations, with interpretation relying on the doctor’s experience, which can lead to misdiagnoses. The 2D imaging from chest X-rays makes it harder to assess lesions, and the high cost and radiation from CT scans also limit their use. In the future, the combination of artificial intelligence and molecular imaging technology could boost diagnostic accuracy and improve early detection and treatment tracking for tuberculosis.

### 5.2. Deep Learning-Based CAD

The application of deep learning technology in the diagnosis of tuberculosis, particularly in the development of CAD systems, has emerged as a key area of research in recent years. Training deep learning algorithms on large amounts of chest X-ray data allows for the automatic identification and localization of tuberculosis lesions, significantly improving the accuracy and efficiency of diagnosis. Deep convolutional neural networks (CNNs) overcome the limitations of traditional manual feature extraction by automatically extracting multi-level features from images, making them particularly suitable for analyzing complex lesion manifestations in chest X-ray or CT images. Pre-trained models such as EfficientNet and DenseNet have become popular in tuberculosis detection and classification tasks, especially when paired with transfer learning and data augmentation techniques, effectively enhancing the model’s generalization ability and robustness [96,97]. Furthermore, deep learning methods which integrate multi-modal features, such as ensemble learning strategies combining manual and deep features, have also significantly improved tuberculosis detection performance [98]. In the analysis of CT images related to bone tuberculosis, deep learning models using multi-modal feature fusion effectively identified characteristics of lesions, such as bone destruction, significantly enhancing diagnostic capabilities [99,100]. Based on large datasets such as TBX11K, the SymFormer model introduces a symmetry attention mechanism (SymAttention) and symmetric positional encoding (SPE), taking full advantage of the bilateral symmetry characteristics of chest X-ray images, significantly improving the detection and classification accuracy of tuberculosis infection areas. The model has achieved the current optimal performance on the TBX11K dataset, signifying a breakthrough in deep learning technology for tuberculosis CAD [101]. The promotion and application of CAD systems, especially in resource-limited primary care and remote areas, hold great promise. Through automated analysis, CAD can effectively alleviate the strain caused by the shortage of specialized radiologists, improving the speed and accuracy of tuberculosis screening and diagnosis, facilitating early detection, and reducing the risk of disease transmission [102,103]. In high-burden tuberculosis countries such as the Philippines and China, CAD systems have been shown to maintain high sensitivity while reducing the workload of radiologists, leading to efficient screening [103,104]. Current deep learning CAD systems for tuberculosis diagnosis are facing challenges, including limitations in the quality and quantity of training datasets, insufficient clinical validation, and limited model transparency. To tackle these issues, it is necessary to construct more diverse datasets, develop integrated learning models, strengthen clinical validation, and enhance model interpretability to improve model generalization capability and build trust among physicians.

## 6. Future Perspectives

### 6.1. Integrated Application of Multimodal Diagnostic Technology

Multimodal diagnostic technology integrates various information from molecular biology, immunological, imaging, and clinical symptoms to construct comprehensive diagnostic models, significantly enhancing the sensitivity and specificity of tuberculosis diagnosis. In recent years, with the integration of big data and artificial intelligence (AI) technologies, machine learning models using multimodal data have shown great potential in predicting tuberculosis diagnosis and treatment outcomes. For instance, large-scale multinational data analysis of patients with multidrug-resistant tuberculosis (MDR-TB) has shown that machine learning models that integrate various information such as radiology, microbiology, treatment regimens, and demographic data can effectively predict treatment outcomes, achieving an accuracy of 83%, outperforming models that rely on a single data type [105]. Furthermore, in HIV/AIDS patients, diagnostic models constructed by combining multimodal imaging genomics with deep learning and clinical features have performed excellently in identifying MDR-TB, with an area under the curve (AUC) of 0.899 in the validation set, significantly outperforming single-modal models [106]. These studies indicate that multimodal diagnostic models that integrate clinical manifestations, molecular testing, and imaging data not only improve the accuracy of early tuberculosis diagnosis but also provide important support for personalized treatment. Multimodal technology has also facilitated the precise classification of different clinical manifestations of tuberculosis. For instance, the joint analysis of single-cell RNA sequencing and surface protein data has shown the variety of immune cell states after tuberculosis infection and their association with disease progression, providing support for a deeper understanding of the immunopathological mechanisms of tuberculosis [107]. Concurrently, multimodal machine learning approaches combining metagenomic sequencing data and host gene expression information can effectively differentiate pulmonary tuberculosis from lung cancer and other infections, enhancing the accuracy of differential diagnosis [108]. AI-assisted multimodal integration systems have shown they can match or even outperform experienced physicians in diagnosing pulmonary infections, quickly identifying different pathogen subtypes, guiding personalized medication, and reducing the risk of antibiotic misuse [109]. Moreover, multimodal diagnostic technology emphasizes the combination of traditional microbiological culture and molecular biological testing to ensure the accuracy and comprehensiveness of resistance testing. Although molecular biological tests such as PCR and GeneXpert are rapid diagnostic tools with high sensitivity and specificity, there remains the possibility of false positives or misjudgments, especially in atypical cases [110,111]. Therefore, the combined use of molecular biological testing and traditional culture methods can complement each other’s limitations, ensuring the accurate identification of resistant strains and helping to create effective treatment plans. Especially in the management of MDR-TB and extensively drug-resistant TB (XDR-TB), combined testing strategies contribute to a comprehensive assessment of the drug susceptibility profile of the strain, optimizing individualized treatment regimens [105].

In summary, multimodal diagnostic technology constructs high-dimensional, intelligent diagnostic models by integrating molecular biology, immunology, imaging, and clinical data, significantly enhancing the sensitivity and specificity of tuberculosis diagnosis while providing scientific evidence for resistance testing and personalized treatment. This layered and diverse diagnostic strategy is becoming a key focus in diagnosing and managing tuberculosis, especially in resource-limited, high-burden areas, with broad application prospects.

### 6.2. Personalized Diagnosis

Non-invasive detection methods based on non-sputum samples, such as blood or urine, are being developed to meet the needs of different patient populations. Traditional sputum testing has limitations in certain patient groups (e.g., children, individuals with AIDS, and those unable to produce sputum), prompting researchers to actively explore tuberculosis diagnostic methods based on non-invasive samples such as blood and urine. Circulating cell-free DNA (cfDNA) in blood, as an emerging biomarker, shows promising application prospects in tuberculosis diagnosis. Studies indicate that detecting MTB-derived cfDNA in plasma can aid in the diagnosis of pulmonary tuberculosis. cfDNA levels correlate with the immune response and show a decreasing trend during treatment, suggesting its potential for disease monitoring [112]. Furthermore, the detection of lipoarabinomannan (LAM) in urine has been recommended by the WHO as an adjunctive diagnostic tool for active tuberculosis in individuals with AIDS. Novel LAM detection technologies, such as FujiLAM, have demonstrated high sensitivity and specificity in the diagnosis of pediatric pulmonary tuberculosis [113,114]. Blood biomarker detection methods developed using nanotechnology and proteomics also offer new avenues for non-invasive and rapid diagnosis [115]. Additionally, integrating multi-omics data with AI models enables precise TB diagnosis and risk prediction, laying the foundation for providing personalized treatment plans in clinical practice [116]. However, current diagnostic methods using non-sputum samples still face challenges such as insufficient sensitivity and a lack of standardized testing protocols, necessitating further clinical validation and technical optimization [114,117]. In the future, interdisciplinary collaboration integrating molecular biology, AI, and clinical medicine will promote the clinical translation of non-invasive detection technologies, benefiting a greater number of tuberculosis patients.

## 7. Conclusions

Tuberculosis is a significant infectious disease worldwide, and advancements in its laboratory diagnostic techniques are directly related to early detection and effective treatment. This study reviews current laboratory diagnostic techniques for tuberculosis, covering the integrated application of traditional diagnostic methods and modern molecular technologies. It provides an in-depth analysis of the advantages and limitations of various techniques in terms of sensitivity, specificity, operational convenience, and clinical applicability (Table 1). Traditional diagnostic methods such as sputum smear microscopy and culture techniques remain the preferred methods in many resource-limited settings due to their simplicity and low cost. However, these methods have limitations in diagnostic efficiency and accuracy, demonstrating suboptimal performance, particularly in detecting latent infections and multidrug-resistant tuberculosis infections. The introduction of modern molecular diagnostic technologies such as Xpert MTB/RIF and gene sequencing technologies, has significantly improved the sensitivity and specificity of detection, enabling rapid and accurate identification of MTB and drug-resistant genes, which holds significant implications for clinical diagnosis and treatment guidance. The development of computer-aided imaging diagnostic technology has provided new possibilities for the rapid screening of tuberculosis. Tuberculosis prevention and control should reasonably allocate diagnostic techniques based on regional medical resources and epidemiological characteristics, achieving complementary use of both traditional methods and modern technologies to ensure both testing accuracy and cost-effectiveness. The future development trends in tuberculosis diagnosis should aim for the integration of multiple technologies. By bringing together the strengths of molecular biology, imaging, and AI, we can maximize diagnostic efficiency and accuracy. At the same time, advancements in diagnostic technology should go hand in hand with personalized medicine, creating tailored treatment plans based on the specific pathogen characteristics and drug resistance of the patient, which helps improve treatment outcomes and cut down on side effects and resistance risks. We are at a crucial point in tuberculosis diagnosis technology, and the ongoing blend of tech innovation with clinical practice will greatly promote the realization of early and accurate diagnosis. Through multidisciplinary collaboration and the integration of resources, it is anticipated that future efforts will substantially reduce diagnostic blind spots and undetected tuberculosis cases, thereby alleviating the disease burden and contributing to the attainment of global objectives for TB prevention and control.

## Figures and Tables

**Table 1:** Comparison and analysis of the advantages and disadvantages, sensitivity, and applicable populations of diagnostic methods for tuberculosis using bacteriology, molecular biology, and immunology.

Category	Techniques	Advantages	Disadvantages	Sensitivity	Applicable Population
Bacterial morphological diagnostic techniques	Sputum Smear Microscopy Examination Technique	low cost, easy to operate, suitable for initial screening.	low sensitivity, prone to missed diagnosis	Z-N acid-fast staining 50–60%, fluorescent staining 70–80%	active tuberculosis
	Bacterial Culture Techniques	the gold standard for diagnosis, high specificity	long duration, easy contamination during the cultivation process	liquid culture about 90%, solid culture 75–80%	active tuberculosis, combined with drug susceptibility tests, can diagnose drug-resistant tuberculosis
Molecular biology diagnosis techniques	Nested PCR, LAMP, and Gene Chip Technique	high sensitivity and specificity, fast detection speed, and the ability to simultaneously identify drug-resistant genes	unable to distinguish between live and dead bacteria, the equipment is expensive, and there are false positives	80–90%, up to 96%	active tuberculosis and drug-resistant tuberculosis
Immunological diagnostic techniques	ELISPOT, IGRA	screen of latent infection of tuberculosis	unable to distinguish between latent infection and active infection	sensitivity varies	latent tuberculosis and active tuberculosis

## Data Availability

No new data were created or analyzed in this review.
